# Supplementary material for: Factor V Leiden, Factor II, Protein C, Protein S, and Antithrombin and Ischemic Strokes in Young Adults: A Meta-Analysis
Source: Genes (Basel). 2022 Nov 9;13(11):2081. doi: 10.3390/genes13112081 (PMC9690045; doi:10.3390/genes13112081)

Supplemental figure 1 Forest plot for FVL, when the studies reported the prevalence of hetero -and homozygotes, subgroup analysis according to equal or unequal gender ratio. All of the included articles are presented with the corresponding number of mutated alleles in the experimental group and the control group, and the total number of alleles. The blue squares indicate the Odds ratio. The black rhombus indicates the overall effect. The vertical line indicates the null effect, and the horizontal line – the value of the odds ratio.

	Experimental		Control			Odds Ratio
Study or Subgroup	Events	Total	Events	Total	Weight	M-H, Fixed, 95% CI
**1.1.5 Equal gender ratio**						
Biswas et al 2009	14	240	1	240	0.6%	14.81 [1.93, 113.51]
Bondarenko et al 2011	31	2032	17	1086	14.9%	0.97 [0.54, 1.77]
Botto et al 2007	2	194	1	320	0.5%	3.32 [0.30, 36.89]
Djordjevic et al 2012	6	146	7	240	3.5%	1.43 [0.47, 4.33]
Haeusler et al 2012	7	88	22	564	3.7%	2.13 [0.88, 5.14]
Karttunen et al 2003	4	114	1	208	0.5%	7.53 [0.83, 68.17]
Madonna et al 2000	7	264	17	524	7.6%	0.81 [0.33, 1.98]
M'barek et al 2020	16	322	7	228	5.3%	1.65 [0.67, 4.08]
Offelli et al 2007	1	152	2	152	1.4%	0.50 [0.04, 5.54]
Pezzini et al 2003	6	250	5	298	3.0%	1.44 [0.43, 4.78]
Pezzini et al 2005	6	326	5	316	3.4%	1.17 [0.35, 3.86]
Ranelloua et al 2014	7	102	4	140	2.1%	2.51 [0.71, 8.80]
Supanc et al 2014	16	310	5	300	3.3%	3.21 [1.16, 8.88]
They-They et al 2011	0	182	0	364		Not estimable
**Subtotal (95% CI)**		**4722**		**4980**	**49.9%**	**1.65 [1.23, 2.20]**
Total events	123		94			
Heterogeneity: Chi² = 15.79, df = 12 (P = 0.20); I² = 24%						
Test for overall effect: Z = 3.38 (P = 0.0007)						
**1.1.6 Unequal gender ratio**						
Amico et al 1998	5	62	4	248	1.0%	5.35 [1.39, 20.56]
Anadure et al 2017	6	240	2	240	1.3%	3.05 [0.61, 15.27]
Chatterjeea et al 2013	0	104	0	104		Not estimable
de Paula Sabinoa et al 2006	4	106	3	550	0.6%	7.15 [1.58, 32.43]
Eterovi´c et al 2005	10	240	3	240	2.0%	3.43 [0.93, 12.64]
Hashemi et al 2018	16	212	13	314	6.6%	1.89 [0.89, 4.02]
Kheradmand et al 2013	0	44	2	108	1.0%	0.48 [0.02, 10.17]
Kumar et al 2017	9	500	5	500	3.4%	1.81 [0.60, 5.45]
Lichy et al 2005	12	154	15	404	5.2%	2.19 [1.00, 4.80]
Longstreth et al 1997	1	210	16	764	4.7%	0.22 [0.03, 1.70]
Martinelli et al 2006	6	210	7	586	2.5%	2.43 [0.81, 7.32]
Narayan et al 2020	0	200	0	150		Not estimable
Pahus et al 2015	16	414	287	8376	17.8%	1.13 [0.68, 1.89]
Pestana et al 2009	4	108	4	268	1.5%	2.54 [0.62, 10.34]
Pirhoushiara et al 2014	4	306	2	306	1.4%	2.01 [0.37, 11.07]
Shi et al 2008	0	194	0	198		Not estimable
Tosetto et al 1997	0	46	3	230	0.8%	0.70 [0.04, 13.76]
Weih et al 1998	3	24	1	72	0.3%	10.14 [1.00, 102.69]
**Subtotal (95% CI)**		**3374**		**13658**	**50.1%**	**1.77 [1.36, 2.31]**
Total events	96		367			
Heterogeneity: Chi² = 18.38, df = 14 (P = 0.19); I² = 24%						
Test for overall effect: Z = 4.19 (P < 0.0001)						
**Total (95% CI)**		**8096**		**18638**	**100.0%**	**1.71 [1.40, 2.08]**
Total events	219		461			
Heterogeneity: Chi² = 34.71, df = 27 (P = 0.15); I² = 22%						
Test for overall effect: Z = 5.33 (P < 0.00001)						
Test for subgroup differences: Chi² = 0.13, df = 1 (P = 0.71), I² = 0%						

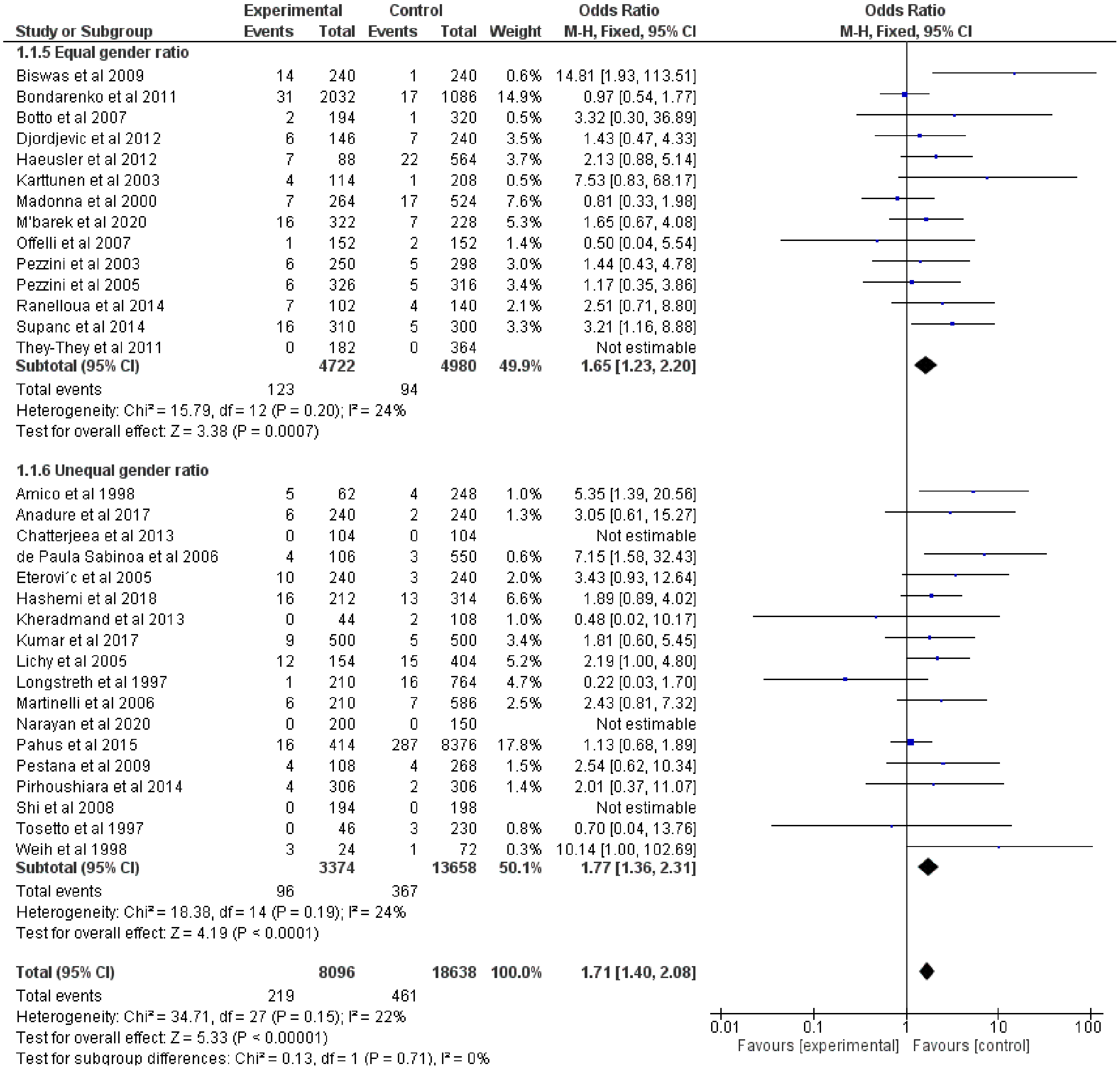

Supplemental figure 2 Forest plot for FVL, when the studies reported the prevalence of hetero -and homozygotes, subgroup analysis according to cryptogenic stroke or non-cryptogenic stroke. All of the included articles are presented with the corresponding number of mutated alleles in the experimental group and the control group, and the total number of alleles. The blue squares indicate the Odds ratio. The black rhombus indicates the overall effect. The vertical line indicates the null effect, and the horizontal line – the value of the odds ratio.

	Experimental		Control			Odds Ratio
Study or Subgroup	Events	Total	Events	Total	Weight	M-H, Fixed, 95% CI
**1.1.3 Cryptogenic stroke**						
Amico et al 1998	5	62	4	248	1.0%	5.35 [1.39, 20.56]
Anadure et al 2017	6	240	2	240	1.4%	3.05 [0.61, 15.27]
Biswas et al 2009	14	240	1	240	0.7%	14.81 [1.93, 113.51]
Chatterjeea et al 2013	0	104	0	104		Not estimable
Eterovi´c et al 2005	10	240	3	240	2.0%	3.43 [0.93, 12.64]
Haeusler et al 2012	7	88	22	564	3.9%	2.13 [0.88, 5.14]
Hashemi et al 2018	16	212	13	314	6.8%	1.89 [0.89, 4.02]
Karttunen et al 2003	4	114	1	208	0.5%	7.53 [0.83, 68.17]
Kheradmand et al 2013	0	44	2	108	1.0%	0.48 [0.02, 10.17]
M'barek et al 2020	16	322	7	228	5.5%	1.65 [0.67, 4.08]
Narayan et al 2020	0	200	0	150		Not estimable
Offelli et al 2007	1	152	2	152	1.4%	0.50 [0.04, 5.54]
Pahus et al 2015	16	414	287	8376	18.3%	1.13 [0.68, 1.89]
Ranelloua et al 2014	7	102	4	140	2.2%	2.51 [0.71, 8.80]
Tosetto et al 1997	0	46	3	230	0.8%	0.70 [0.04, 13.76]
Weih et al 1998	3	24	1	72	0.3%	10.14 [1.00, 102.69]
**Subtotal (95% CI)**		**2604**		**11614**	**45.8%**	**2.00 [1.51, 2.63]**
Total events	105		352			
Heterogeneity: Chi² = 17.60, df = 13 (P = 0.17); I² = 26%						
Test for overall effect: Z = 4.90 (P < 0.00001)						
**1.1.4 Non-cryptogenic stroke**						
Bondarenko et al 2011	31	2032	17	1086	15.4%	0.97 [0.54, 1.77]
Botto et al 2007	2	194	1	320	0.5%	3.32 [0.30, 36.89]
de Paula Sabinoa et al 2006	4	106	3	550	0.7%	7.15 [1.58, 32.43]
Djordjevic et al 2012	6	146	7	240	3.6%	1.43 [0.47, 4.33]
Kumar et al 2017	9	500	5	500	3.5%	1.81 [0.60, 5.45]
Lichy et al 2005	12	154	15	504	4.6%	2.75 [1.26, 6.02]
Longstreth et al 1997	1	105	16	764	2.7%	0.45 [0.06, 3.42]
Madonna et al 2002	7	264	17	524	7.8%	0.81 [0.33, 1.98]
Martinelli et al 2006	6	210	7	586	2.5%	2.43 [0.81, 7.32]
Pestana et al 2009	4	108	4	268	1.6%	2.54 [0.62, 10.34]
Pezzini et al 2003	6	250	5	298	3.1%	1.44 [0.43, 4.78]
Pezzini et al 2005	6	326	5	316	3.5%	1.17 [0.35, 3.86]
Pirhoushiara et al 2014	4	306	2	306	1.4%	2.01 [0.37, 11.07]
Shi et al 2008	0	194	0	198		Not estimable
Supanc et al 2014	16	310	5	300	3.4%	3.21 [1.16, 8.88]
They-They et al 2011	0	182	0	364		Not estimable
**Subtotal (95% CI)**		**5387**		**7124**	**54.2%**	**1.58 [1.19, 2.09]**
Total events	114		109			
Heterogeneity: Chi² = 15.62, df = 13 (P = 0.27); I² = 17%						
Test for overall effect: Z = 3.17 (P = 0.002)						
**Total (95% CI)**		**7991**		**18738**	**100.0%**	**1.77 [1.45, 2.16]**
Total events	219		461			
Heterogeneity: Chi² = 33.54, df = 27 (P = 0.18); I² = 19%						
Test for overall effect: Z = 5.64 (P < 0.00001)						
Test for subgroup differences: Chi² = 1.39, df = 1 (P = 0.24), I² = 27.9%						

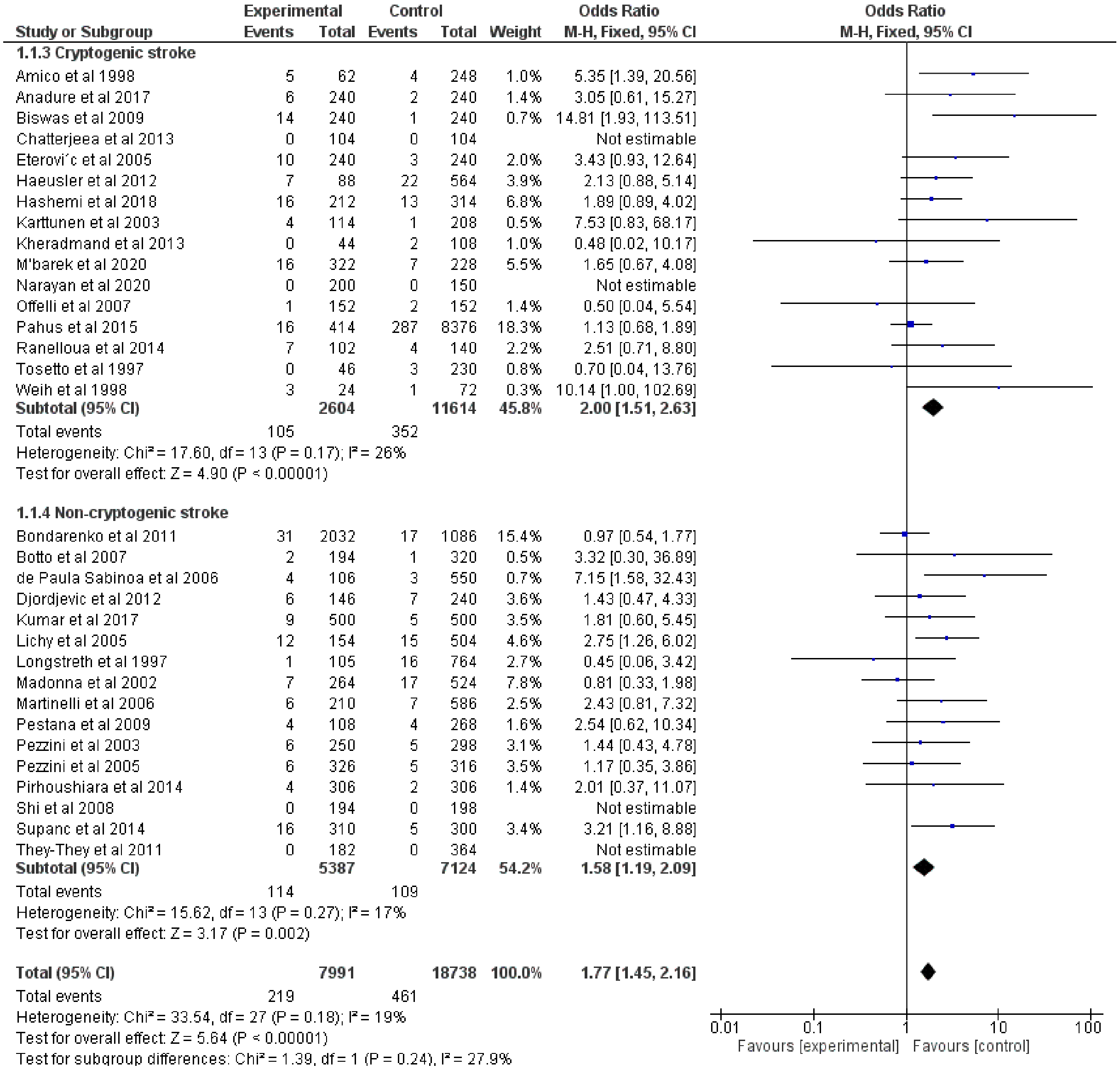

Supplemental figure 3 Forest plot for FVL, when the studies reported their participants as mutants and healthy individuals only. The blue squares indicate the Odds ratio. The black rhombus indicates the overall effect. The vertical line indicates the null effect, and the horizontal line – the value of the odds ratio.

	Experimental		Control			Odds Ratio
Study or Subgroup	Events	Total	Events	Total	Weight	M-H, Fixed, 95% CI
Ashjazadeh et al 2012	4	57	0	50	0.5%	8.50 [0.45, 161.82]
Aznarl et al 2004	2	49	5	294	1.4%	2.46 [0.46, 13.05]
Hamedani et al 2013	30	830	34	907	31.8%	0.96 [0.58, 1.59]
Lalouschek et al 2005	29	53	30	52	13.9%	0.89 [0.41, 1.92]
Pongrácz et al 2007	8	189	4	173	4.1%	1.87 [0.55, 6.32]
Rodrigues et al 2004	2	42	3	134	1.4%	2.18 [0.35, 13.53]
Sastry et al 2006	4	101	8	101	7.8%	0.48 [0.14, 1.65]
Slooter et al 2005	14	179	42	763	14.9%	1.46 [0.78, 2.73]
Tasdemir et al 2016	99	599	8	100	11.6%	2.28 [1.07, 4.84]
Urbanus et al 2009	11	190	33	767	12.5%	1.37 [0.68, 2.76]
**Total (95% CI)**		**2289**		**3341**	**100.0%**	**1.30 [1.00, 1.69]**
Total events	203		167			

Heterogeneity: Chi² = 9.89, df = 9 (P = 0.36); I² = 9%
Test for overall effect: Z = 1.99 (P = 0.05)

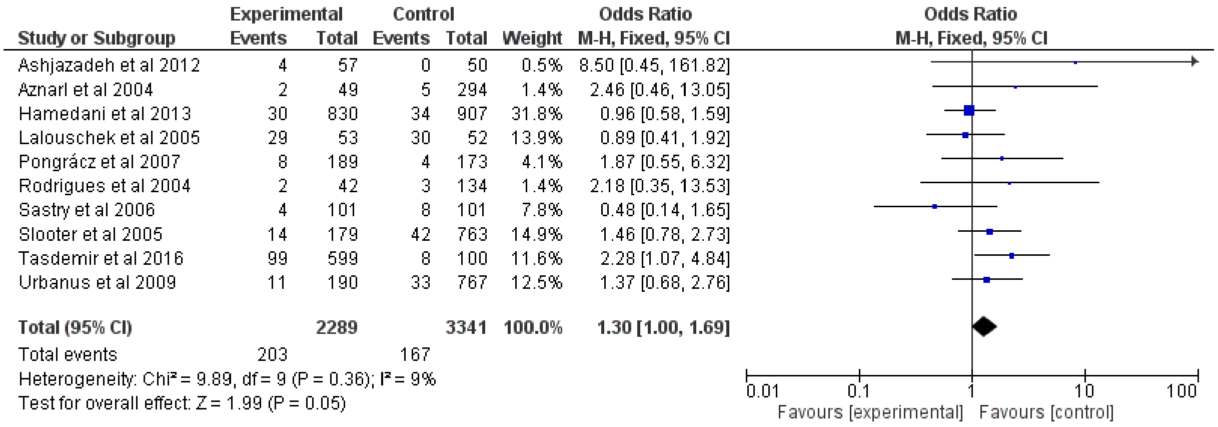

Supplemental figure 4 Forest plot for prothrombin, when the studies reported their participants as mutants and healthy individuals only, and subgroup analysis for prothrombin, based on the geographic location of the study. The blue squares indicate the Odds ratio. The black rhombus indicates the overall effect. The vertical line indicates the null effect, and the horizontal line – the value of the odds ratio.

	Experimental		Control			Odds Ratio
Study or Subgroup	Events	Total	Events	Total	Weight	M-H, Fixed, 95% CI
**1.2.1 Europe**						
Lalouschek et al 2005	23	468	9	468	30.5%	2.64 [1.21, 5.76]
Pongrácz et al 2007	89	189	2	173	3.9%	76.09 [18.34, 315.74]
Slooter et al 2005	5	188	18	763	24.7%	1.13 [0.41, 3.09]
Tasdemir et al 2016	47	599	2	100	11.3%	4.17 [1.00, 17.46]
Urbanus et al 2009	3	190	15	767	20.9%	0.80 [0.23, 2.81]
**Subtotal (95% CI)**		**1634**		**2271**	**91.3%**	**5.17 [3.52, 7.59]**
Total events	167		46			
Heterogeneity: Chi² = 33.97, df = 4 (P < 0.00001); I² = 88%						
Test for overall effect: Z = 8.39 (P < 0.00001)						
**1.2.2 Asia and South America**						
Ashjazadeh et al 2012	2	57	2	50	7.3%	0.87 [0.12, 6.43]
Rodrigues et al 2004	7	42	1	134	1.4%	26.60 [3.17, 223.41]
**Subtotal (95% CI)**		**99**		**184**	**8.7%**	**5.04 [1.46, 17.44]**
Total events	9		3			
Heterogeneity: Chi² = 5.31, df = 1 (P = 0.02); I² = 81%						
Test for overall effect: Z = 2.56 (P = 0.01)						
**Total (95% CI)**		**1733**		**2455**	**100.0%**	**5.16 [3.57, 7.44]**
Total events	176		49			

Heterogeneity: Chi² = 39.25, df = 6 (P < 0.00001); I² = 85%
Test for overall effect: Z = 8.77 (P < 0.00001)
Test for subgroup differences: Chi² = 0.00, df = 1 (P = 0.97), I² = 0%

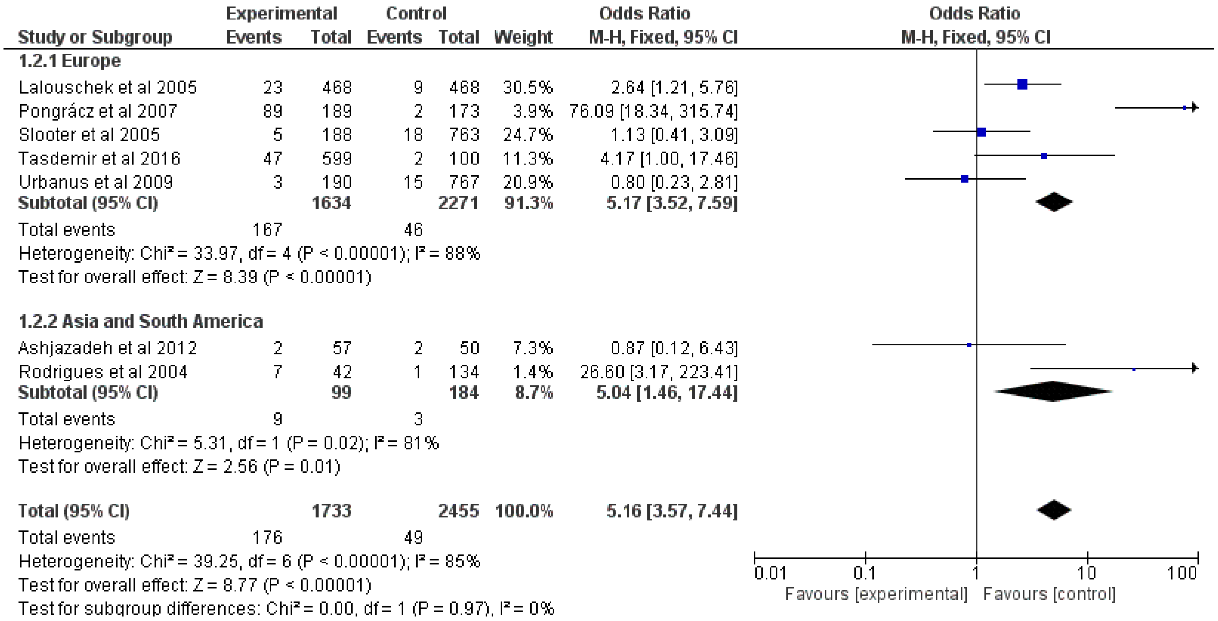

Supplemental figure 5 Forest plot for prothrombin, when the studies reported the prevalence of hetero -and homozygotes, subgroup analysis for equal or unequal gender ratio. All of the included articles are presented with the corresponding number of mutated alleles in the experimental group and the control group, and the total number of alleles. The blue squares indicate the Odds ratio. The black rhombus indicates the overall effect. The vertical line indicates the null effect, and the horizontal line – the value of the odds ratio.

	Experimental		Control			Odds Ratio
Study or Subgroup	Events	Total	Events	Total	Weight	M-H, Fixed, 95% CI
**1.1.3 Equal gender ratio**						
Biswas et al 2009	0	240	0	240		Not estimable
Bondarenko et al 2011	16	2032	9	1086	16.6%	0.95 [0.42, 2.16]
Botto et al 2007	8	194	3	320	3.1%	4.54 [1.19, 17.34]
Djordjevic et al 2012	4	146	5	240	5.2%	1.32 [0.35, 5.01]
Garcia et al 2002	5	98	4	174	3.9%	2.28 [0.60, 8.72]
Karttunen et al 2003	2	114	0	208	0.5%	9.27 [0.44, 194.69]
Madonna et al 2002	10	264	16	524	14.7%	1.25 [0.56, 2.79]
Offelli et al 2007	1	152	2	152	2.8%	0.50 [0.04, 5.54]
Pezzini et al 2003	5	250	3	298	3.8%	2.01 [0.47, 8.48]
Pezzini et al 2005	10	326	3	316	4.2%	3.30 [0.90, 12.11]
Ranellou et al 2014	2	102	5	140	5.9%	0.54 [0.10, 2.84]
Supanc et al 2014	7	310	2	300	2.8%	3.44 [0.71, 16.70]
They-They et al 2011	11	182	10	364	8.9%	2.28 [0.95, 5.47]
**Subtotal (95% CI)**		**4410**		**4362**	**72.5%**	**1.72 [1.23, 2.42]**
Total events	81		62			
Heterogeneity: Chi² = 11.18, df = 11 (P = 0.43); I² = 2%						
Test for overall effect: Z = 3.13 (P = 0.002)						
**1.1.4 Unequal gender ratio**						
Chatterjeea et al 2013	0	104	0	104		Not estimable
de Paula Sabino et al 2006	10	106	0	550	0.2%	119.80 [6.96, 2061.27]
Eterovi´c et al 2005	9	240	3	240	4.1%	3.08 [0.82, 11.51]
Hashemi et al 2018	0	212	0	314		Not estimable
Jiang et al 2014	0	794	0	852		Not estimable
Lichy et al 2005	8	154	5	404	3.7%	4.37 [1.41, 13.58]
Longstreth et al 1997	2	210	6	764	3.7%	1.21 [0.24, 6.06]
Martinelli et al 2006	5	210	15	586	11.0%	0.93 [0.33, 2.59]
Pirhoushiaran et al 2014	0	186	2	306	2.7%	0.33 [0.02, 6.84]
Saadatnia et al 2012	0	44	2	108	2.1%	0.48 [0.02, 10.17]
**Subtotal (95% CI)**		**2260**		**4228**	**27.5%**	**2.57 [1.60, 4.13]**
Total events	34		33			
Heterogeneity: Chi² = 15.47, df = 6 (P = 0.02); I² = 61%						
Test for overall effect: Z = 3.89 (P < 0.0001)						
**Total (95% CI)**		**6670**		**8590**	**100.0%**	**1.95 [1.48, 2.58]**
Total events	115		95			
Heterogeneity: Chi² = 26.79, df = 18 (P = 0.08); I² = 33%						
Test for overall effect: Z = 4.73 (P < 0.00001)						
Test for subgroup differences: Chi² = 1.80, df = 1 (P = 0.18), I² = 44.5%						

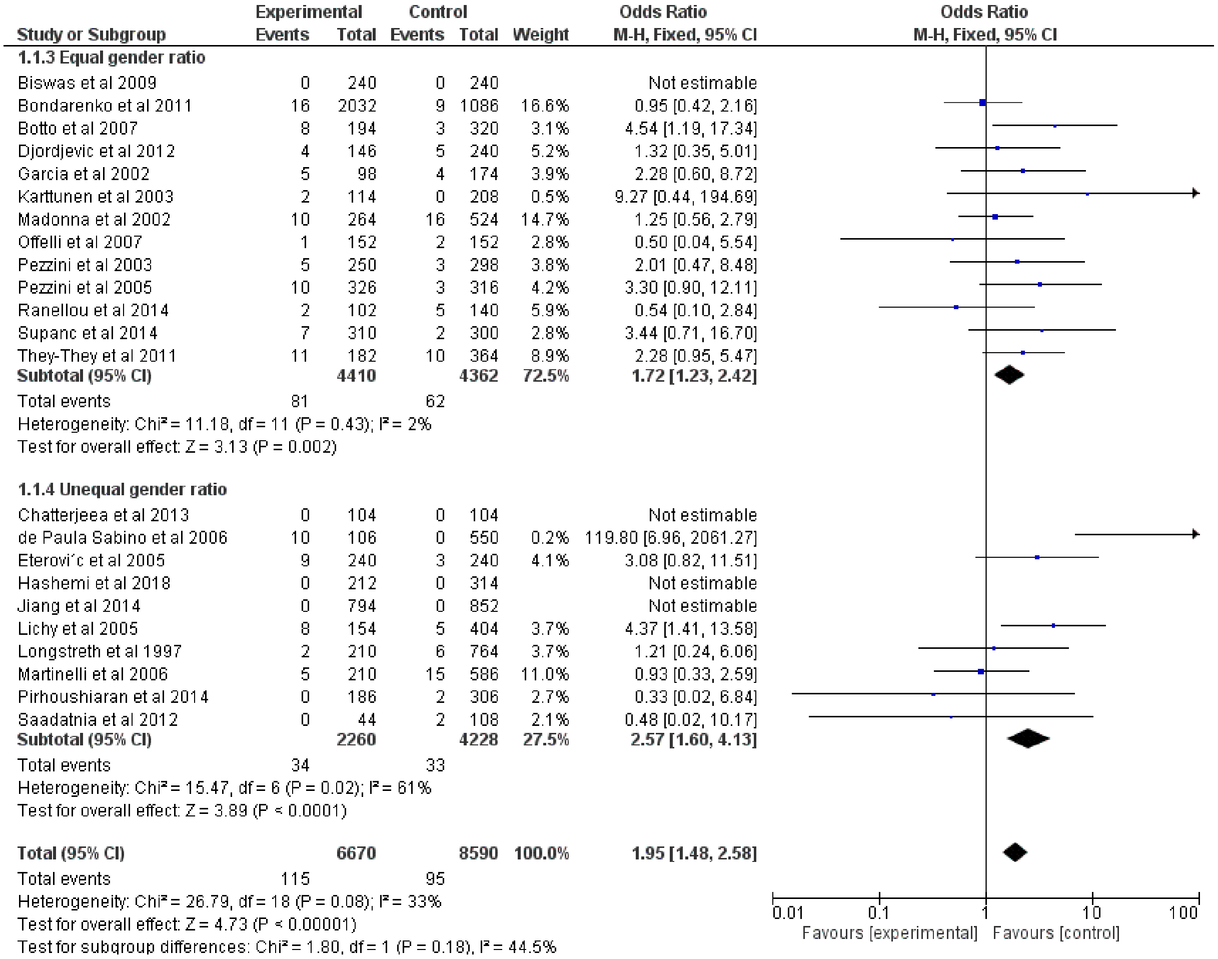

Supplemental figure 6 Forest plot for prothrombin, when the studies reported the prevalence of hetero -and homozygotes, subgroup analysis for cryptogenic and non-cryptogenic stroke. All of the included articles are presented with the corresponding number of mutated alleles in the experimental group and the control group, and the total number of alleles. The blue squares indicate the Odds ratio. The black rhombus indicates the overall effect. The vertical line indicates the null effect, and the horizontal line – the value of the odds ratio.

	Experimental		Control			Odds Ratio
Study or Subgroup	Events	Total	Events	Total	Weight	M-H, Fixed, 95% CI
**1.1.1 Cryptogenic stroke**						
Biswas et al 2009	0	240	0	240		Not estimable
Chatterjeea et al 2013	0	104	0	104		Not estimable
Eterovi´c et al 2005	9	240	3	240	4.1%	3.08 [0.82, 11.51]
Garcia et al 2002	5	98	4	174	3.9%	2.28 [0.60, 8.72]
Hashemi et al 2018	0	212	0	314		Not estimable
Karttunen et al 2003	2	114	0	208	0.5%	9.27 [0.44, 194.69]
Offelli et al 2007	1	152	2	152	2.8%	0.50 [0.04, 5.54]
Ranellou et al 2014	2	102	5	140	5.9%	0.54 [0.10, 2.84]
Saadatnia et al 2012	0	44	2	108	2.1%	0.48 [0.02, 10.17]
**Subtotal (95% CI)**		**1306**		**1680**	**19.3%**	**1.65 [0.85, 3.19]**
Total events	19		16			
Heterogeneity: Chi² = 5.64, df = 5 (P = 0.34); I² = 11%						
Test for overall effect: Z = 1.47 (P = 0.14)						
**1.1.2 Non-crytpogenic stroke**						
Bondarenko et al 2011	16	2032	9	1086	16.6%	0.95 [0.42, 2.16]
Botto et al 2007	8	194	3	320	3.1%	4.54 [1.19, 17.34]
de Paula Sabino et al 2006	10	106	0	550	0.2%	119.80 [6.96, 2061.27]
Djordjevic et al 2012	4	146	5	240	5.2%	1.32 [0.35, 5.01]
Jiang et al 2014	0	794	0	852		Not estimable
Lichy et al 2005	8	154	5	404	3.7%	4.37 [1.41, 13.58]
Longstreth et al 1997	2	210	6	764	3.7%	1.21 [0.24, 6.06]
Madonna et al 2002	10	264	16	524	14.7%	1.25 [0.56, 2.79]
Martinelli et al 2006	5	210	15	586	11.0%	0.93 [0.33, 2.59]
Pezzini et al 2003	5	250	3	298	3.8%	2.01 [0.47, 8.48]
Pezzini et al 2005	10	326	3	316	4.2%	3.30 [0.90, 12.11]
Pirhoushiaran et al 2014	0	186	2	306	2.7%	0.33 [0.02, 6.84]
Supanc et al 2014	7	310	2	300	2.8%	3.44 [0.71, 16.70]
They-They et al 2011	11	182	10	364	8.9%	2.28 [0.95, 5.47]
**Subtotal (95% CI)**		**5364**		**6910**	**80.7%**	**2.03 [1.49, 2.75]**
Total events	96		79			
Heterogeneity: Chi² = 21.17, df = 12 (P = 0.05); I² = 43%						
Test for overall effect: Z = 4.54 (P < 0.00001)						
**Total (95% CI)**		**6670**		**8590**	**100.0%**	**1.95 [1.48, 2.58]**
Total events	115		95			
Heterogeneity: Chi² = 26.79, df = 18 (P = 0.08); I² = 33%						
Test for overall effect: Z = 4.73 (P < 0.00001)						
Test for subgroup differences: Chi² = 0.32, df = 1 (P = 0.57), I² = 0%						

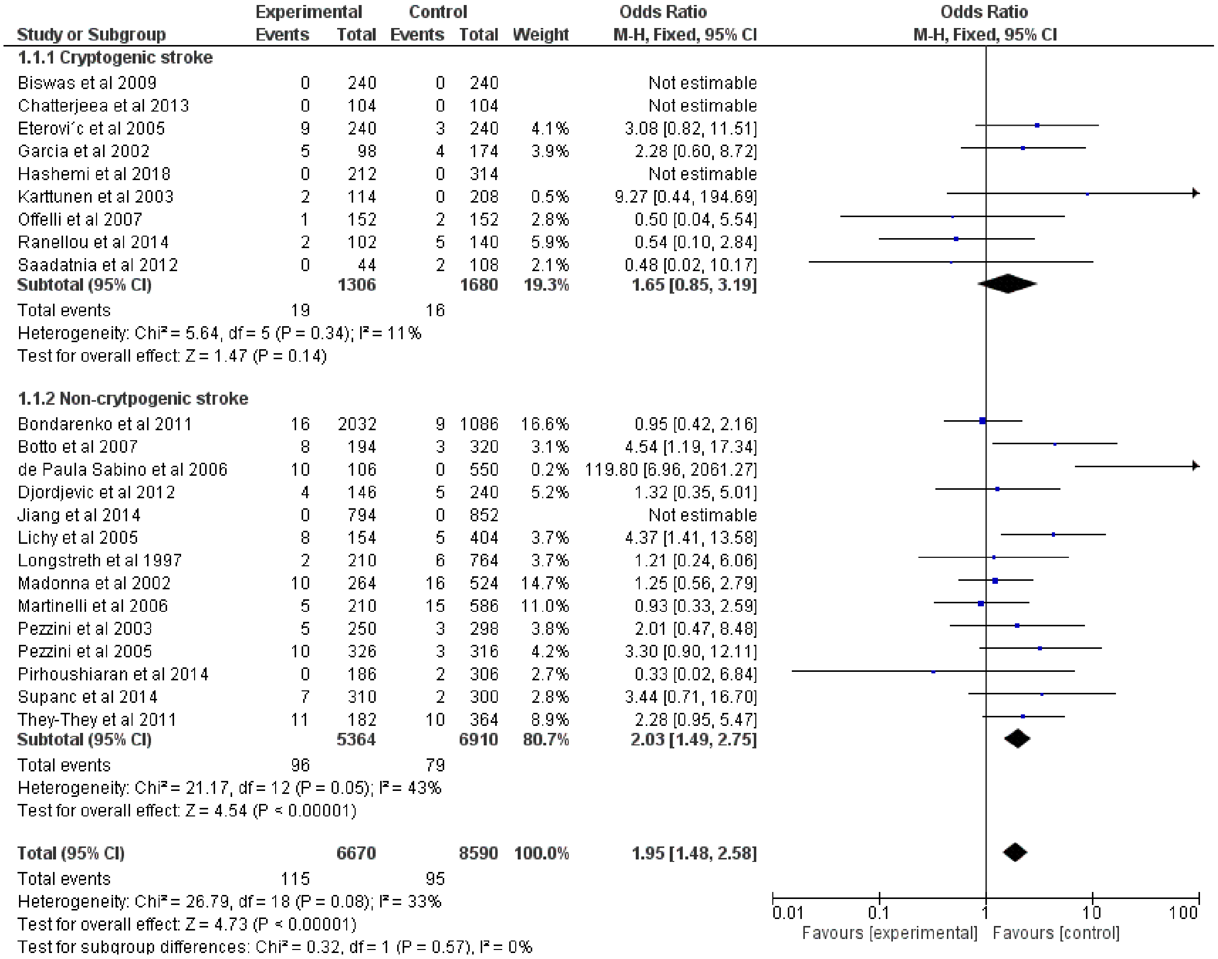

Supplemental figure 7 Forest plot for Antithrombin and subgroup analysis according to gender ratio of the studies. The blue squares indicate the Odds ratio. The black rhombus indicates the overall effect. The vertical line indicates the null effect, and the horizontal line – the value of the odds ratio.

Study or Subgroup	Experimental Events	Experimental Total	Control Events	Control Total	Weight	Odds Ratio M-H, Fixed, 95% CI
**1.1.3 Equal gender ratio**						
Karttunen et al 2003	1	58	2	104	8.9%	0.89 [0.08, 10.08]
Mojiminiyi et al 2008	0	102	0	167		Not estimable
Sastry et al 2006	3	101	1	101	6.1%	3.06 [0.31, 29.94]
**Subtotal (95% CI)**		**261**		**372**	**15.0%**	**1.78 [0.37, 8.46]**
Total events	4		3			
Heterogeneity: Chi² = 0.53, df = 1 (P = 0.47); I² = 0%						
Test for overall effect: Z = 0.72 (P = 0.47)						
**1.1.4 Unequal gender ratio**						
Chatterjeea et al 2013	0	52	0	52		Not estimable
Jerrard-Dunne et al 2003	0	130	0	130		Not estimable
Narayan et al 2020	41	100	18	75	76.7%	2.20 [1.13, 4.27]
Pahus et al 2015	0	288	16	9669	6.0%	1.01 [0.06, 16.94]
Stepien et al 2019	3	83	16	9669	1.7%	22.62 [6.47, 79.17]
Tosetto et al 2003	0	23	0	0		Not estimable
Zeidman et al 2003	9	68	0	604	0.6%	193.03 [11.10, 3358.07]
**Subtotal (95% CI)**		**744**		**20199**	**85.0%**	**3.77 [2.20, 6.46]**
Total events	53		50			
Heterogeneity: Chi² = 18.52, df = 3 (P = 0.0003); I² = 84%						
Test for overall effect: Z = 4.82 (P < 0.00001)						
**Total (95% CI)**		**1005**		**20571**	**100.0%**	**3.47 [2.09, 5.76]**
Total events	57		53			
Heterogeneity: Chi² = 19.97, df = 5 (P = 0.001); I² = 75%						
Test for overall effect: Z = 4.80 (P < 0.00001)						
Test for subgroup differences: Chi² = 0.80, df = 1 (P = 0.37), I² = 0%						

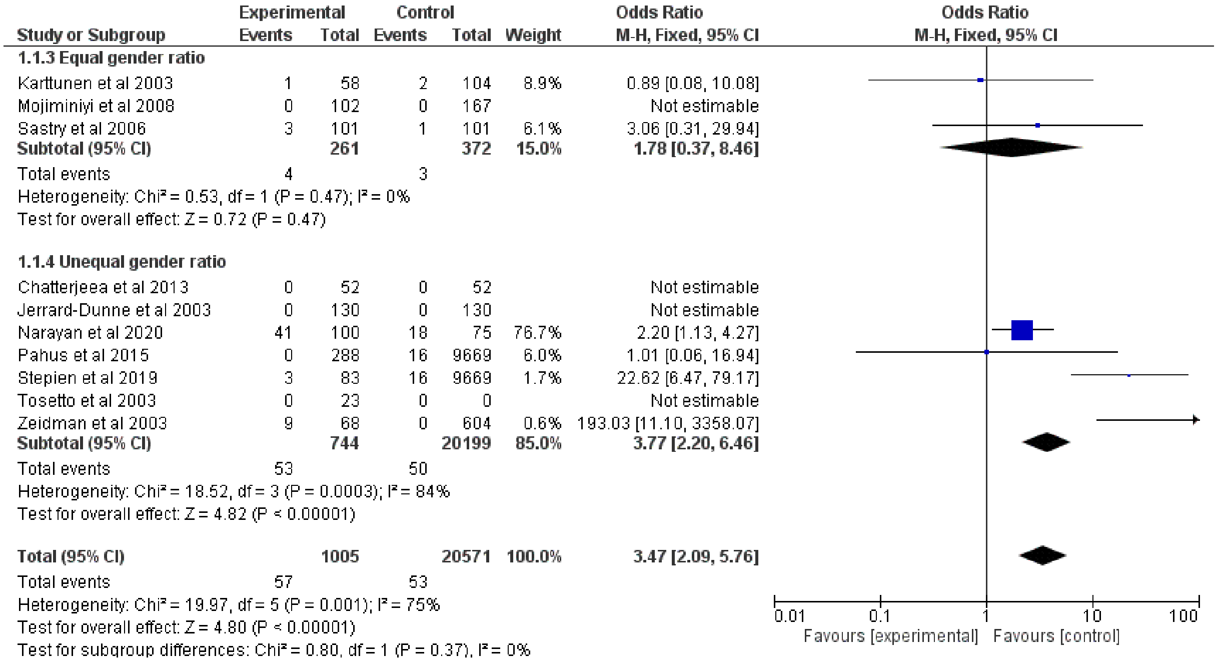

Supplemental figure 8 Funnel plots for all five meta-analyses: A - Factor V Leiden; B – Prothrombin; C - Protein C; D - Protein S; E – Antithrombin.

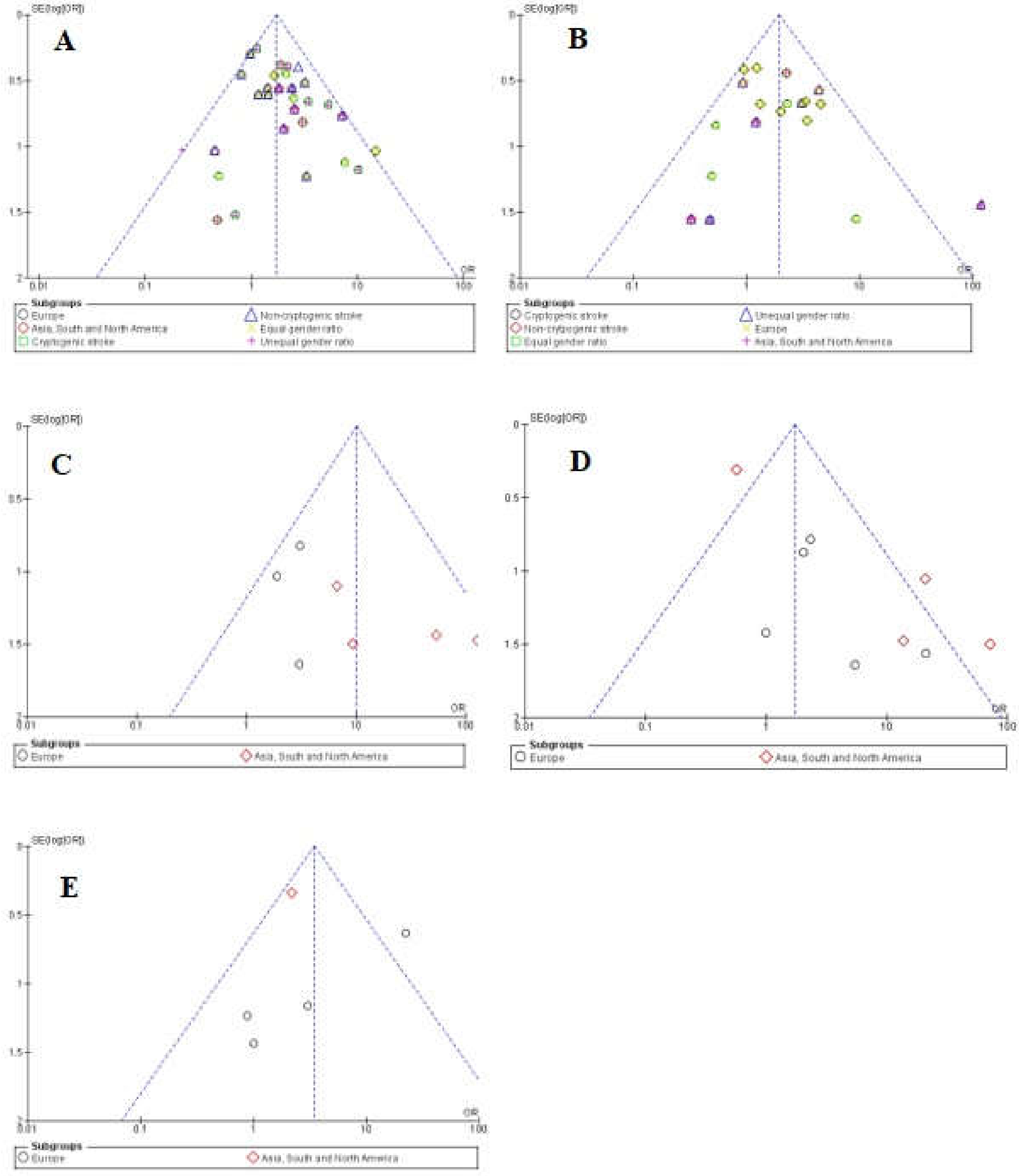

Supplement: Supplementary file 1 [file genes-13-02081-s001.zip › Supplemental figures.pdf]
